# Yao-Shan of traditional Chinese medicine: an old story for metabolic health

**DOI:** 10.3389/fphar.2023.1194026

**Published:** 2023-08-16

**Authors:** Shuangling Yang, Hongzhi Yang, Yaxing Zhang

**Affiliations:** ^1^ School of Health Sciences, Guangzhou Xinhua University, Guangzhou, Guangdong, China; ^2^ Department of Traditional Chinese Medicine, The Third Affiliated Hospital, Sun Yat-Sen University, Guangzhou, Guangdong, China; ^3^ Department of Physiology, School of Basic Medical Sciences, Guangzhou University of Chinese Medicine, Guangzhou, Guangdong, China; ^4^ Research Centre of Basic Integrative Medicine, School of Basic Medical Sciences, Guangzhou University of Chinese Medicine, Guangzhou, Guangdong, China; ^5^ Key Laboratory of Chinese Medicine Pathogenesis and Therapy Research, School of Basic Medical Sciences, Guangzhou University of Chinese Medicine, Guangzhou, Guangdong, China

**Keywords:** Chinese Yao-Shan, diabetes, nonalcoholic fatty liver diseases, hyperuricemia, gout, sexual dysfunction

## Abstract

Type 2 diabetes mellitus, nonalcoholic fatty liver disease (NAFLD), cardio-cerebrovascular diseases (CCVDs), hyperuricemia and gout, and metabolic-related sexual dysfunction are metabolic diseases that affect human health in modern society. Scientists have made great efforts to investigate metabolic diseases using cell models *in vitro* or animal models in the past. However, the findings from cells or animals are difficult to translate into clinical applications due to factors such as the *in vitro* and *in vivo* differences; the differences in anatomy, physiology, and genetics between humans and animals; and the differences in microbiome–host interaction. The Chinese have extensively used the medicated diet of traditional Chinese medicine (TCM) (also named as Yao-Shan of TCM, Chinese Yao-Shan et al.) to maintain or improve cardiometabolic health for more than 2,200 years. These ancient classic diets of TCM are essential summaries of long-term life and clinical practices. Over the past 5 years, our group has made every effort to collect and sort out the classic Yao-Shan of TCM from the ancient TCM literature since *Spring and Autumn and Warring States Period*, especially these are involved in the prevention and treatment of metabolic diseases, such as diabetes, NAFLD, CCVDs, hyperuricemia and gout, and sexual dysfunction. Here, we summarized and discussed the classic Yao-Shan of TCM for metabolic diseases according to the time recorded in the ancient literature, and revised the Latin names of the raw materials in these Yao-Shan of TCM. Moreover, the modern medicine evidences of some Yao-Shan of TCM on metabolic diseases have also been summarized and emphasized in here. However, the exact composition (in terms of ratios), preparation process, and dosage of many Yao-Shan are not standardized, and their main active ingredients are vague. Uncovering the mystery of Yao-Shan of TCM through modern biological and chemical strategies will help us open a door, which is ancient but now looks new, to modulate metabolic homeostasis and diseases.

## Introduction

Modern human society is encumbered by a pandemic of chronic diseases and conditions in which metabolic dysregulation plays a key role in the pathogenesis and progression (Newgard, 2017). These metabolic disorders may include obesity, type 2 diabetes mellitus (T2DM), nonalcoholic fatty liver disease (NAFLD), cardio-cerebrovascular diseases (CCVDs), hyperuricemia and gout, metabolic-related sexual dysfunction, and complications associated with these diseases, which are collectively referred to as cardiometabolic diseases (CMDs) (Shi et al., 2015; Zhang et al., 2017a; Zhang et al., 2017b; Zhang et al., 2017c; Zhang and Li, 2017; Dehlin et al., 2020; Chen X. W. et al., 2021; Osborn et al., 2021; Zhang et al., 2021; Angulo and Hannan, 2022; Choi et al., 2022; Nappi et al., 2022; Zhang et al., 2022). Among CMDs, CCVDs remain the leading cause of disease burden worldwide (Roth et al., 2020). For example, there are about 330 million patients with CCVDs in China, which account for 46.74% and 44.26% of all deaths occurring in China’s rural and urban areas: two out of five deaths were caused by CCVDs. The total hospitalization costs in China were 313.366 billion RMB for CCVDs in 2019 (China Cardiovascular Health and Disease, 2022). NAFLD, which is viewed as the hepatic manifestation of metabolic syndrome, is associated with obesity and encompasses a broad spectrum of conditions, from simple steatosis [always refers to nonalcoholic fatty liver (NAFL)], through nonalcoholic steatohepatitis (NASH), to fibrosis and cirrhosis, and ultimately hepatocellular carcinoma (HCC) (Samuel and Shulman, 2018; Zhang Y et al., 2020; Loomba et al., 2021). Moreover, NAFLD and T2DM frequently coexist as they share the same pathogenic abnormalities of excess adiposity and insulin resistance (Smith and Adams, 2011). Currently, 25% of the world population is thought to have NAFLD (Younossi et al., 2019; Lazarus et al., 2022), and the national prevalence of NAFLD in China has exceeded 29% (Zhou et al., 2019; Zhou et al., 2020). The estimated annual medical costs directly attributable to NAFLD have exceeded $103 billion in the United States and €35 billion in four European countries (Germany, France, Italy, and the United Kingdom) (Zhou et al., 2020). The pandemic of NAFLD fuels the upsurge in cardiovascular diseases (CVDs), and currently, NAFLD is emerging as an essential driver for CVDs, such as atherosclerosis, hypertension, and cardiac arrhythmia (Cai et al., 2020; Zhang L et al., 2020; Zhao et al., 2020; Chen Z. et al., 2021; Zhou et al., 2021; Li et al., 2022). CVDs are the leading cause of death in patients with NAFLD (Lazarus et al., 2022). In addition, metabolism dysfunction is recognized as a major contributor to diseases untraditionally considered “metabolic” in origin, such as cancer, cognitive disorders, and respiratory pathologies (Newgard, 2017). CMDs are difficult for physicians to manage because CMDs can be present for years before becoming clinically apparent (Roberts and Gerszten, 2013). Therefore, discovering the accurate predictors of CMDs, and prevention and treatment strategies, are of particular importance.

Although great efforts have been made by biological and medical scientists using cell or animal models to investigate the pathogenesis and treatment strategies for CMDs, these findings appear difficult to translate into humans due to the *in vitro* and *in vivo* differences; the differences between humans and animals in anatomy, physiology, genetics, and the differences in gut (also including vagina and others) microbiome–host interaction, among other factors (Łaniewski et al., 2020; Jardon et al., 2022). Currently, prevention or delay of the morbidity of CMDs is possible via pharmacological and behavioral interventions (e.g., weight control and diet modification) (Roberts and Gerszten, 2013; Morgan and Singh, 2021). However, most patients always do not accept long-term drug-lowering therapies due to a lack of compliance and, thus, exhibit a poor treatment response. Alternatively, they increasingly prefer non-classical pharmacological interventions (Morgan and Singh, 2021). Therefore, it will be beneficial if the daily diet has medicinal properties. This will not only satisfy human demand for material and energy, human yearning for delicious food, and use for social entertainment but, more importantly, also improve the quality of life and maintain human health. In the past 5 years, we have made every effort to collect and sort out medical diets from the ancient classic traditional Chinese medicine (TCM) literature. Here, we summarize the TCM diets for metabolic diseases according to the time of their recording in TCM literature. We also discuss the research progress of these diets using modern biological strategies. We hope these can provide beneficial references for the prevention and treatment of modern metabolic diseases.

## The medicated diet in traditional Chinese medicine

The Chinese have a special form of diet containing Chinese medicinal materials, which achieves the purposes of nutritional value, health preservation, and prevention and treatment of human diseases. Therefore, such diets have several names, such as Yao-Shan of TCM, Yao-Shan in TCM, TCM Yao-Shan, medicated diet in/of TCM, TCM diet, Chinese medicated diet, Chinese Yao-Shan, and ancient classic diet of TCM (Wang, 1987; Yen et al., 2008; Deng, 2010; Li H W et al., 2010). One form of Yao-Shan of TCM frequently found in China is the preparation of medical plants, such as Gouqizi {also as Goji Berry, *Lycium barbarum* L. [Solanaceae; Lycii fructus], *Lycium chinense* Mill. [Solanaceae; Lycii fructus]}, Danggui {*Angelica sinensis* (Oliv.) Diels [Apiaceae; Angelicae sinensis radix]}, and Fuling {*Poria cocos* (Schw.) Wolf [Polyporaceae; Poria]}, in Chinese cuisine and beverages (e.g., porridge, soup, and tea) (Yen et al., 2008).

Yao-Shan of TCM had been originally created by the Chinese for more than 2200 years; it is an important part of TCM. The word “Yao-Shan” was first described in “*Hou Han Shu · Lie Nv Zhuan*-74,” dating back to before 445. Several early formed Yao-Shan of TCM have been recorded in medical literature unearthed from Ma-Wang-Dui Han Tombs in Changsha, China, such as “*Yang Sheng Fang*,” “*Za Liao Fang*,” “*Tai Chan Shu*,” and “*Wu Shi Er Bing Fang*,” which were formed before 168 BC. In ancient China, the government of each dynasty had officials in charge of medicated diet. For example, the department in the central government of *Tang Dynasty* specialized in supplying Yao-Shan for the imperial court was called “Shan Bu,” and the department in charge of Yao-Shan in the central government of *Qing Dynasty* was called “Jing Shan Si.”

Yao-Shan of TCM has been extensively used by the Chinese in daily healthcare and in the treatment of human diseases based on the TCM theoretical system (e.g., diabetes, NAFLD, and other metabolic disorders). Moreover, Yao-Shan of TCM has wildly spread abroad, greatly impacting Japan, the Korean Peninsula, and the Association of Southeast Asian Nations.

## Yao-Shan of TCM for Xiao-Ke (and/or diabetes)

Diabetes mellitus primarily includes type 1 diabetes (T1D) and T2MD in modern medicine (Sims et al., 2021). T1D is a chronic autoimmune disease caused by the immune-mediated destruction of pancreatic *β* cells, resulting in lifelong absolute insulin deficiency (Atkinson et al., 2014; Warshauer et al., 2020). T2MD is characterized by relative insulin deficiency caused by pancreatic *β*-cell dysfunction and insulin resistance in target organs. The main drivers of T2DM are the rise in obesity, a sedentary lifestyle, an energy-dense diet, and population aging (Chatterjee et al., 2017; Zheng et al., 2018). Of the estimated 463 million adults with diabetes mellitus globally, >90% have T2DM, of which approximately 50% live in two large countries: India and China (Ke et al., 2022).

**FIGURE 1:**
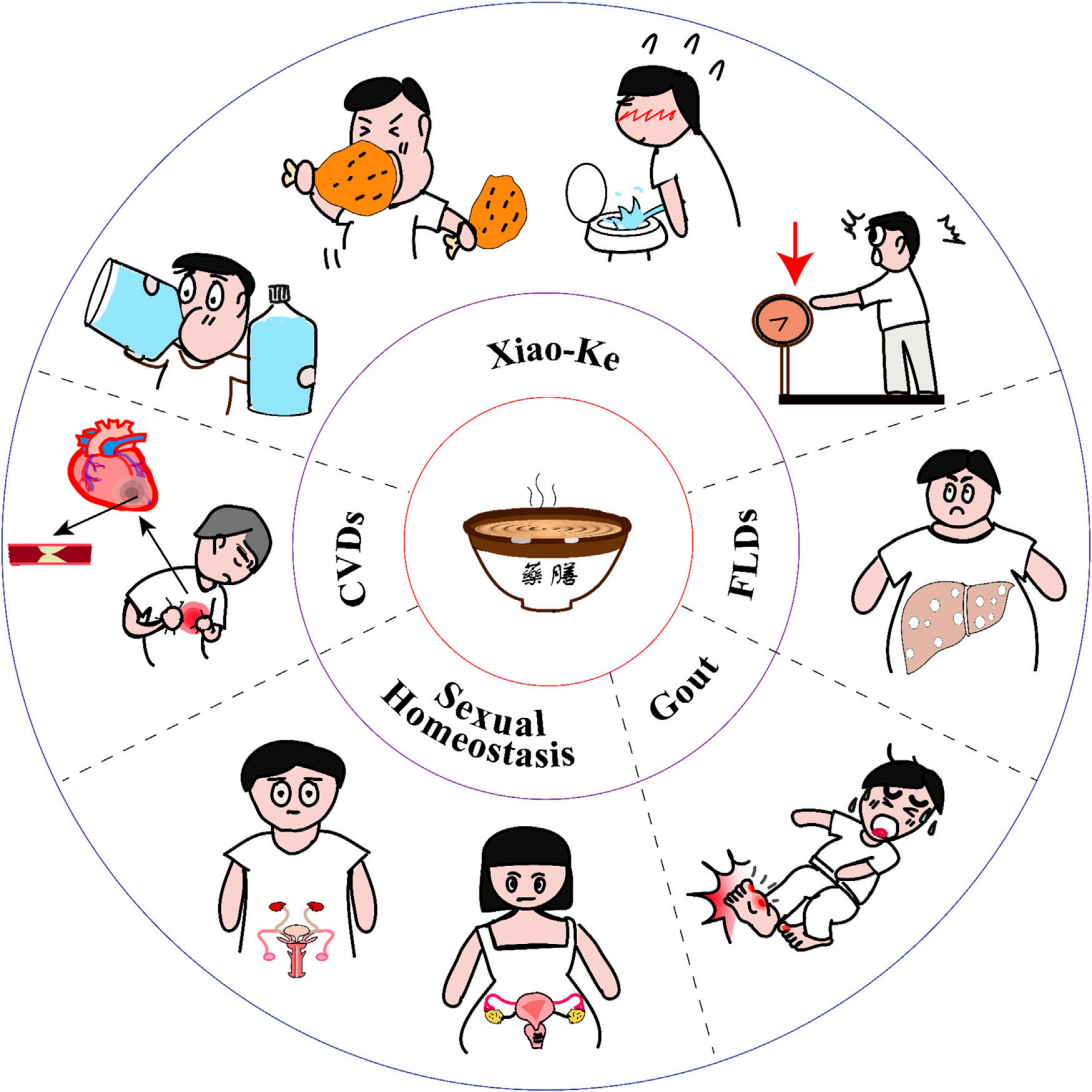
Yao-Shan of traditional Chinese medicine (Yao-Shan of TCM) for metabolic health and diseases. The traditional Yao-Shan of TCM always includes soup, congee, and drinks. They have been used for treating Xiao-Ke, which is characterized by excessive urination, excessive drinking water, excessive diet consumption, and weight loss; fatty liver diseases (FLDs, including NAFLD and ALD); cardiovascular diseases (CVDs); hyperuricemia and gout; and male and female sexual dysfunction. However, prescribing a Yao-Shan of TCM is more complicated than prescribing a pill. It should be prescribed and prepared with the guidance of the TCM theory and the cooking theory. Yao-Shan of TCM will open a door, which is ancient but now looks new, to modulate metabolic homeostasis and diseases.

In TCM, diabetes mellitus belongs to the category of Xiao-Ke and others. Xiao-Ke was first recorded in *Qi-Bing-Lun* Section 47 from the classic TCM literature *Huang Di Nei Jing · Su Wen* (Wang and Cheng, 1999; Chen et al., 2002; Wu, 2002; Lv, 2006; Chen et al., 2015; Tong, 2016). It has been considered that the symptoms of Xiao-Ke are characterized by excessive urination, excessive drinking water, excessive diet consumption (the regular food that a person eats each day), and weight loss. All these are collectively referred to as “three excessive and one loss” (Tong et al., 2012; Chen et al., 2015) (Figure 1). Turbid or sweet urine is also an essential feature of Xiao-Ke (Tong et al., 2012; Chen et al., 2015). The main pathogenesis of Xiao-Ke is yin deficiency and endogenous dryness-heat in body, these two always mutually influence each other, the above two can be induced by unhealthy diet, emotional disorder, and excessive sexual activities (Tong et al., 2012; Chen et al., 2015; Pang et al., 2015). If prolonged yin deficiency impairs yang, dual deficiency of qi (“qi” is a broad TCM concept, generally refers to “the most basic substances that maintain human life activities”) and yin, as well as dual deficiency of yin and yang, will occur (Pang et al., 2015). In addition, blood stasis is also involved in the pathogenesis of Xiao-Ke. Therefore, the basic therapeutic methods for Xiao-Ke are invigorating qi (supplement qi), nourishing yin, clearing heat, and promoting fluid production (generating body fluids) (Pang et al., 2015).

Since ancient times, the Chinese have realized that the combination of some daily feeding diets may cause Xiao-Ke. *Huang Di Shi Jin* described that eating “Fanlou {also as Fanluo, *Stellaria media* (L.) Vill. [Caryophyllaceae]}” with “Yu-Shan-Zha (a kind of salted fish)” can induce Xiao-Ke. *Yang Sheng Yao Ji* written by Zhang Zhan in the *Chinese East Jin* (*or North Wei*) *Dynasty* revealed that eating Yu-Zha (also as a kind of salted fish) with Dou (it refers to beans, however, the details are unclear) may induce Xiao-Ke. Similarly, *Sun Zhen Ren Shi Ji*, written by Sun Simiao in the *Chinese Tang Dynasty*, further described that eating Chidou {also as Chixiaodou, *Vigna angularis* (Willd.) Ohwi & H.Ohashi [Fabaceae; Vignae semen], *Vigna umbellata* (Thunb.) Ohwi & H.Ohashi [Fabaceae; Vignae semen]} or Baidou (the plant and its corresponding Latin name it refers to are yet to be verified) combined with Yuzha may also induce Xiao-Ke. Both *San Yuan Can Zan Yan Shou Shu* (written by Li Pengfei in the *Chinese Yuan Dynasty*) and *Shi Jin Fang* reported that “eating the roast meat of Chinese water deer {*Hydropotes inermis* Swinhoe [Cervidae]} may cause Xiao-Ke.” *San Yuan Can Zan Yan Shou Shu* also showed that “eating uncooked dog meat may cause Xiao-Ke.” Similar to this, *Ben Cao Yue Yan*, written by Xue Ji in the *Chinese Ming Dynasty*, stated that “eating the roast meat of yellow dog may cause Xiao-Ke.” Importantly, *Xue Ji* also revealed that alcohol may cause Xiao-Ke. Zhang Zhan had showed that “eating wheat with Gumi {the caryopsis of *Zizania latifolia* (Griseb.) Turcz. ex Stapf. [Gramineae]}, then, drinking alcohol will lead to Xiao-Ke”. Li Pengfei had described “if an unawakened drunk feel great thirst, then, he drinks cold water and cold tea, cold will be introduced into the kidney by the wine, and become cold poison; if much and long, it will cause heavy of waist and knees (and foot), cold pain of bladder, edema, Xiao-Ke and Luanbi”. This was also reported by Wu Zhenglun from the *Chinese Ming Dynasty* in *Yang Sheng Lei Yao*. However, it is not clear whether the above factors can cause Xiao-Ke in humans, who live in modern society. If so, what is the mechanism behind these changes, and further exploration is needed.

In contrast, the Chinese had used ancient classic TCM prescriptions, or even a single Chinese medicinal material, and the TCM diets to treat Xiao-Ke for more than 2,200 years, for example, *Huang Di Nei Jing · Su Wen* showed Xiao-Ke can be treated by “Lan {it may refer to Peilan, *Eupatorium fortunei* Turcz. [Asteraceae]}” through its ability to eliminate “Chen Qi”. Here, we summarize and discuss these ancient classic TCM diets according to their publishing time. Some of these discussed ancient classic TCM diets have been recorded repeatedly in the following ancient literature, indicating that they may undergo more clinical practices.

*Shen Nong Ben Cao Jing*, the earliest materia medica literature in China, reported that Gouqizi, the white parts of excrement from red cock, Baiying {*Solanum lyratum* Thunb. [Solanaceae]}, Gegen {*Pueraria lobata* (Willd.) Ohwi [Fabaceae; Puerariae lobatae radix]}, Gualougen {also as “Tianhuafen”, *Trichosanthes kirilowii* Maxim. [Cucurbitaceae; Trichosanthis radix], *Trichosanthes rosthornii* Harms [Cucurbitaceae; Trichosanthis radix]}, Zhimu {*Anemarrhena asphodeloides* Bunge [Asparagaceae; Anemarrhenae rhizoma]}, Fuping {also as “Shuiping”, *Lemna minor* L. [Araceae]}, Wanggua {also named as “Tugua” (Shen, 1993; Zhang, 1994), *Trichosanthes cucumeroides* (Ser.) Maxim. ex Franch. & Sav. [Cucurbitaceae], *Thladiantha dubia* Bunge [Cucurbitaceae]} and Sumi {the mature seeds of *Setaria italica* (L.) P.Beauv. [Poaceae]} were the representative Yao-Shan for alleviating Xiao-Ke. The polysaccharides and flavonoids in Gouqizi (Masci et al., 2018; Yang et al., 2022; Zhu et al., 2022), polysaccharide and puerarin in Gegen (Wong et al., 2011; Wang et al., 2020; Luo D et al., 2021), mangiferin in Zhimu (Li X et al., 2010), and TK protein in Gualougen (Lo et al., 2017) are the key antidiabetic bioactive molecules.

*Qi Juan Shi Jing* described that Gugen {rhizome and root of *Zizania latifolia* (Griseb.) Turcz. ex Stapf. [Gramineae]} and Fanlou may improve Xiao-Ke. However, we do not know why eating Fanlou with Yu-Shan-Zha may induce Xiao-Ke, whereas eating Fanlou alone is beneficial for Xiao-Ke. As Yu-Shan-Zha is a regular diet in China, it is urgent to answer the mechanisms of these differences.

*Cui Yu Xi Shi Jing*, written by Cui Yuxi in the *Chinese Jin Dynasty*, reported that Kuicai {*Malva verticillata* L. [Malvaceae]} (Wei, 1964; Ma and Wang, 2016), Shichun {*Ulva lactuca* L. [Ulvaceae]}, Zitai (it may refer to a type of algae), Haiyue {the meat of *Placuna placenta* L. [Placunidae]}, Wuyu {it may refer to Biqi, *Eleocharis dulcis* (Burm.f.) Trin. ex Hensch. [Cyperaceae]; or Cigu, *Sagittaria trifolia* L. [Alismataceae], (Sun et al., 2013)}, Mihoutao {*Actinidia chinensis* Planch. [Actinidiaceae]}, the bamboo shoot {always refers to the seedling of *Pleioblastus amarus* (Keng) Keng f. [Poaceae], *Phyllostachys glauca* McClure [Poaceae], *Phyllostachys edulis* (Carrière) J.Houz. [Poaceae]}, the deer’s head and meat, Shi-Yin-Zi, Long-Ti-Zi, and He-Bei-Zi (however, the biological or mineral sources of the later three still need further investigation), were the representative Yao-Shan for alleviating Xiao-Ke. Some of these Chinese Yao-Shan for Xiao-Ke were further confirmed by modern medicine. The polysaccharides from *Ulva lactuca* L. [Ulvaceae] (ULP, 180 mg/kg per day for 30 days by oral gavage) improve antioxidant balance and bone mineral density in alloxan-induced diabetic Wistar rat model, and the low dose of ULP (100 mg/kg) significantly alleviated hyperglycemia and glucose tolerance impairment in diabetic mice caused by D-gal and streptozotocin (STZ) with a high-sugar, high-fat diet (Sahla et al., 2021; Chen et al., 2022). One clinical trial showed that drinking fruit juice of *Actinidia chinensis* Planch. [Actinidiaceae] (10 ml per day) for nine months, and combined with exercise (each patient walks about 1.5 to 2 miles per day), improves anti-oxidative and anti-inflammatory status of T2DM patients by activating Kelch-like ECH-associated protein 1 (Keap1) and nuclear factor erythroid-derived 2-like 2 (Nrf2) via increasing microRNA-424 (Sun et al., 2017). Feeding the basal diet mixed with 5% (w/w) dietary fibers from bamboo shoot shells to diabetic mice induced by HFD plus STZ for four weeks display the hypoglycemic effect (Zheng et al., 2019). The β-pyran polysaccharides from bamboo shoot shells (400 mg/kg per day for 3 weeks by oral gavage) may be responsible for this hypoglycemic activity (Zheng et al., 2016). Although there are no direct experimental data of the deer’s head or meat on diabetes, the 5–10 kDa peptides from red deer {*Cervus elaphus* L. [Cervidae]} antler (intraperitoneally, 75, 150, or 300 μg/kg per day for 6 weeks) showed hypoglycemic, hypolipidemic, and antioxidant effects in diabetic mice induced by STZ (Jiang et al., 2015; Wang W et al., 2019). In addition, a new bioactive peptide CPU2206 from sika {*Cervus nippon* Temminck [Cervidae]} antler also alleviated diabetes in alloxan-induced diabetic mice and in obese mice with spontaneous diabetes (KK-Ay mice) (CPU2206, intraperitoneally, 30 or 100 mg/kg; once a day for 28 days in the first animal model, and once a day for 14 days in the second animal model) (Jiang et al., 2015; Wang W et al., 2019). These two studies have confirmed the anti-diabetes effect of the peptides from the antlers. However, antlers and its products are rarely administered by intraperitoneal injection in the clinical practice of TCM. The antlers are often administered orally, so it is more meaningful to study antlers from the perspective of oral administration in future.

*Sun Zhen Ren Shi Ji* stated that soaking the meat of Xian {a species of small clam living in fresh water, it may refer to *Corbicula fluminea* Müller [Corbiculidae]} in water and drinking it has a therapeutic effect on Xiao-Ke. The antidiabetic components of *Corbicula fluminea* Müller [Corbiculidae] include phytosterols and fatty acids (Huang et al., 2022a), a polysaccharide–protein complex (Wang Y Y et al., 2019), and peptides (Huang et al., 2022b). Clinical data showed that consumption of 2 g of the *Corbicula fluminea* Müller [Corbiculidae] extract daily for 180 days reduces serum TNF-*α* levels in pre-diabetic patients in the Taiwan province of China (Huang et al., 2022a). Similar to *Shen Nong Ben Cao Jing*, *Sun Zhen Ren Shi Ji* described Fuping as a benefit for Xiao-Ke.

*Shi Liao Ben Cao*, written by Meng Shen in the *Chinese Tang Dynasty*, reported that Yangru {milk from *Capra hircus* L. [Bovidae], *Ovis aries* L. [Bovidae]}; Niudu {stomach of *Bos taurus domesticus* Gmelin [Bovidae], *Bubalus bubalis* L. [Bovidae]}; Kuige {it might refer to *Arca inflata* Reeve [Arcidae]}, Dongxinggen {also as Linqingen, the root of *Malus asiatica* Nakai. [Rosaceae]}, Pugua {also as Baidonggua, it may refers to *Benincasa hispida* (Thunb.) Cogn. [Cucurbitaceae]}, and Song {refers to *Brassica rapa* L. [Brassicaceae], *Raphanus sativus* L. [Brassicaceae] (Xie, 1994)} are the representative Yao-Shan of TCM for Xiao-Ke. The goat milk improves glucose homeostasis in the diabetic rat model induced by HFD (294.4 g goat milk powder per kg HFD, 24 weeks), STZ (10 g/kg goat milk powder per day for 30 days), or HFD plus STZ (10 ml/kg per day for 5 weeks, oral gavage), partially via activation of hepatic AMP-activated protein kinase (AMPK) (Liu et al., 2019; Liu W et al., 2021; Han B et al., 2022). The extracts from *Raphanus sativus* L. [Brassicaceae] (400 mg/kg per day for 3 weeks by oral gavage) have antidiabetic effects in HFD plus STZ-induced diabetic rat model (Jani and Goswami, 2020). This beneficial effect seems to be attributed to its bioactive compounds, such as flavonoids, saponins, glycosides, and tannins (Jani and Goswami, 2020). Additionally, *Meng Shen* stated that Gouqigen {the root of *Lycium barbarum* L. [Solanaceae], *Lycium chinense* Mill. [Solanaceae]}, bamboo, and Mi-Hou-Tao have therapeutic effects on Xiao-Ke.

*Meng Shen Shi Jing* reported that the boiled juice (or soup) of Mazi {the mature seeds of *Cannabis sativa* L. [Cannabaceae] with shell}; the boiled juice (or soup) of the shell of Chinese chestnut {the fruits of *Castanea mollissima* Blume [Fagaceae]}; the boiled juice (or soup) of Lüdou {the mature seeds of *Phaseolus radiatus* L. [Fabaceae]}; and the soup of the meat from head of donkey have antidiabetic effects on Xiao-Ke. Clinical data showed that cannabis use was associated with a lower risk of diabetes in chronic hepatitis C-infected patients (Barré et al., 2020)*.* Orally administrated phenolic extracts from burs of Chinese chestnut at doses of 150 or 300 mg/kg twice a day for 12 days exhibited potential antidiabetic activities in STZ-induced diabetic rats (Yin et al., 2011). In addition, the former antidiabetic Yao-Shan, such as Wuyu, Linqin, and the meat of deer’s head, were also reported in *Meng Shen Shi Jing*.

*Shi Yi Xin Jing*, written by Zan Yin in the *Chinese Tang Dynasty*, reported that milk of cow {*Bos taurus domesticus* Gmelin [Bovidae], *Bubalus bubalis* L. [Bovidae]}, the white pigeon, Zhi (pheasant), Xiaomai {the mature seeds of *Triticum aestivum* L. [Poaceae]}, the soup of the head (with bones) of rabbit, the soaking water from Tian-Zhong-Luo {*Cipangopaludina chinensis* Gray [Viviparidae]}, and the juice of Laifu {also as Luobo, *Raphanus sativus* L. [Brassicaceae]} were essential Yao-Shan for Xiao-Ke. In animals, the juice of the root of *Raphanus sativus* L. [Brassicaceae] (300 mg/kg, a single oral treatment) showed a hypoglycemic effect in STZ-induced diabetes in Wistar rats (Shukla et al., 2011). Flavonoid-rich wheatgrass {*Triticum aestivum* L. [Poaceae]; used wheatgrass extract at 9^th^ days after germination, 200 or 400 mg/kg per day for 60 days} diet attenuated diabetes induced by STZ plus HFD in Wistar rats (Adhikary et al., 2021). Sulfated polysaccharides (CCPS) from *Cipangopaludina chinensis* Gray [Viviparidae] (100 or 400 mg/kg per day for 12 weeks) attenuated atherosclerosis in high fat diet (HFD)-fed ApoE^−/−^ mice (Xiong et al., 2019). However, its effects on diabetes are unclear, and the water soaked in *Cipangopaludina chinensis* Gray [Viviparidae] might not contain contents such as CCPS, therefore, the mechanisms of Tian-Zhong-Luo on Xiao-Ke still need further investigation. Additionally, Zan Yin also described bamboo shoots and Sumi for Xiao-Ke, which have already been reported in the earlier Yao-Shan literature.

In addition to *Huang Di Nei Jing*, *Shen Nong Ben Cao Jing*, *San Yuan Can Zan Yan Shou Shu*, *Yang Sheng Lei Yao*, and *Ben Cao Yue Yan*, the original texts of other above TCM literature have been lost. The details of some above Yao-Shan were reprinted in *Bei Ji Qian Jin Yao Fang* by Sun Simiao in the *Chinese Tang Dynasty, Zheng He Ben Cao* by Tang Shenwei and *Yang Sheng Lei Zuan* by Zhou Shouzhong both in the *Chinese South Song Dynasty, Shi Jian Ben Cao* by Fei Boxiong in the *Chinese Qing Dynasty*. Many ancient Japanese medicine was learned from China, some of these original texts were also partially reprinted in *Wo Ming Lei Ju Chao* by Yuan Shun (Japanese) in the middle of 10th century of Japan, *Yi Xin Fang* formed in 984 AD by Dan Bo Kang Lai (Japanese), *Wei Sheng Mi Yao Chao* formed in 1228 AD by Dan Bo Xing Zhang (Japanese), and *Wan An Fang* formed in 1315 AD by Wei Yuan Xing Quan (Japanese).

*Bei Ji Qian Jin Yao Fang* declared that Damai {the mature seeds of *Hordeum vulgare* L. [Poaceae]}, Tianhu {the fruits of *Lagenaria siceraria* var. *hispida* (Thunb.) H.Hara [Cucurbitaceae]}, Qing-Liang-Mi {the mature seeds of *Setaria italica* (L.) P.Beauv. [Poaceae], or *Setaria italica* (L.) Beauv. var. germanica (Mill.) Schred. [Poaceae]; if the mature seeds stored for a long time, they called “Chen-Su-Mi”}, Qing-Xiao-Dou (it may refer to Lüdou), Sha-Niu-Sui (the bone marrow of female cattle), the meat of female cattle, and the meat of yellow hen are the representative Yao-Shan for Xiao-Ke. The hydroalcoholic extract of *Hordeum vulgare* L. [Poaceae] (0.25, 0.5 g/kg per day for 11 days by oral gavage) and the seeds extract of *Setaria italica* (L.) P.Beauv. [Poaceae] (300 mg/kg per day for 30 days by oral gavage) have antidiabetic effects in STZ-induced diabetic rats, and the antidiabetic effect of the seeds extract of *Setaria italica* (L.) P.Beauv. [Poaceae] (300 mg/kg per day for 3 weeks) has also been confirmed in HFD plus STZ-induced diabetic rats (Sireesha et al., 2011; Minaiyan et al., 2014; Singh et al., 2022). Supplement of *Hordeum vulgare* L. [Poaceae] (25%, 45%, or 65% whole grain highland barley in diets) for 8 weeks displayed antidiabetic effects in *db/db* mice (Deng et al., 2020). In healthy Japanese, the consumption of a meal containing refined barley (refers to Damai) flour bread is associated with a lower postprandial blood glucose concentration after a second meal compared with one containing refined wheat flour bread (Matsuoka et al., 2020). Bottle gourd {*Lagenaria siceraria* (Molina) Standl. [Cucurbitaceae]} is advocated for diabetes in Ayurveda. However, toxic cucurbitacins in bitter bottle gourd should be considered (Verma and Jaiswal, 2015). Furthermore, *Bei Ji Qian Jin Yao Fang* described the former reported Yao-Shan, including Shichun, Wuyu, Song, bamboo shoot, and the meat from deer’s head, for Xiao-Ke.

*Tai Ping Sheng Hui Fang*, written by Wang Huaiyin et al. in the *Chinese North Song Dynasty*, described many Yao-Shan for Xiao-Ke. Here, we discussed seven Yao-Shan with the specific Chinese name. (1). Yin Shui Bu Zhi Fang (“Fang” refers to the TCM formula or prescription) mainly includes Huangdan (refers to red lead, *Plumbum Rubrum* Minium), Gualougen, Congbai {bulbs of *Allium fistulosum* L. [Amaryllidaceae] near the root}, wheat flour, and Xiebai {*Alliurn macrostemon* Bunge [Amaryllidaceae; Allii macrostemonis bulbus], *Allium chinense* G. Don [Amaryllidaceae; Allii macrostemonis bulbus]}. (2). Gualougen Geng Fang mainly includes Gualougen, Donggua, and Chizhi (refers to the fermented soybean juice). (3). Gua-Lou-Fen Fang (Gua-Lou-Fen refers to Tianhuafen). (4). Xinglao Zhou Fang (Zhou in Yao-Shan of TCM always refers to congee) mainly includes Xinglao, which refers to the porridge of stir apricot {the fruits of *Prunus armeniaca* L. [Rosaceae]} kernels, the milk of cow, Damai, and the white granulated sugar can also be added. (5). Yang-Fei Geng Fang includes the Yangfei {lung of *Capra hircus* L. [Bovidae], Ovis aries L. [Bovidae]}, mutton, the polished round-grained rice {the mature seeds of *Oryza sativa* L. [Poaceae]}, Congbai, ginger, salt, and vinegar et al. (6). Huang-Ci-Ji Zhou Fang (Huang-Ci-Ji refers to the meat of yellow hen). (7). Shen Xiao Zhu Tu Fang (Tu refers to rabbit) mainly includes the rabbit and the fresh Sangbaipi {*Morus alba* L. [Moraceae]}. Moreover, this literature also described the deer’s head, Zhi, Tian-Zhong-Luo, and Luobo, for alleviating Xiao-Ke, and these have always been reported in the earlier Yao-Shan literatures. Although the cooking methods of above Chinese Yao-Shan have been mentioned in *Tai Ping Sheng Hui Fang*, however, further clinical and animal experiments are still needed to confirm whether they are suitable for treating Xiao-Ke in modern society.

*Feng Qin Yang Lao Shu*, written by Chen Zhi in the *Chinese North Song Dynasty*, described Niuru Fang (Niuru refers to the milk of cow), Qing-Liang-Mi Yin Fang, Qingdou Fang (Qingdou may refer to Lüdou), Donggua Geng Fang, Lutou Fang (Lutou refers to the deer’s head), Zhudu Fang (Zhudu refers to the pig stomach), and Lugen Yin Fang {Lugen refer to the rhizome of *Phragmites communis* Trin. [Poaceae]}, were used for alleviating Xiao-Ke. These Yao-Shan were mainly used by drinking juice or eating them after cooking. The key ingredients in the former five Fangs have already been discussed above. Lugen (ethanol extract, 5.0 g/kg per day for 5 weeks; polysaccharide solution of Lugen, 100 mg/kg per day for 3 weeks; given orally by gavage) has antidiabetic effects in STZ-induced diabetic mice (Zhang et al., 2011; Cui et al., 2012; Xu et al., 2012; Song et al., 2014; Zheng et al., 2017). The seven Yao-Shan above have also been reported by Hong Pian from the *Chinese Ming Dynasty* in *Shi Zhi Yang Lao Fang*.

*Sheng Ji Zong Lu*, written by Song Huizong Government in the *Chinese North Song Dynasty*, reported more than twelve Fangs as the representative Yao-Shan of TCM for alleviating Xiao-Ke. (1). Yanggu Tang Fang includes the sheep spine, Chi, the white Sumi and Xiebai. (2). Tianluo Yin Fang. (3). Lüdou Zhi Fang. (4). Hudou Zhi Fang, “Hudou” had been used to refer to Wandou {*Pisum sativum* L. [Fabaceae]} or Candou {*Vicia faba* L. [Fabaceae]} in the ancient times of China, however, it is unclear which specific type is in here. (5). Dihuanghua Zhou Fang, Dihuanghua refers to the follower of *Rehmannia glutinosa* (Gaertn.) DC. [Plantaginaceae]. (6). Liangmi Zhou Fang, “Liangmi” refers to “Qing-Liang-Mi”. (7). Kui Ji Zhi Fang, “Kui” may refer to “*Malva verticillata var. crispa* L. [Malvaceae]”. (8). Mai Dou Yin Fang includes Damai and Lüdou. (9). Gefen Fan Fang includes the powders of Gegen and Sumi. (10). Ou Mi Jiang Fang includes the roots of *Nelumbo nucifera* Gaertn. [Nelumbonaceae] and Fengmi {*Apis cerana* Fabricius [Apidae; Mel], *Apis mellifera* L. [Apidae; Mel]}. (11). Gujianggen Geng Fang includes Gujianggen (Gugen) and Donggua. (12). Yan Chi Yin Fang includes salts and Chizhi. Niuru and Lutou were also described in *Sheng Ji Zong Lu*. In addition, added Oushi {also named as Lianzi, *Nelumbo nucifera* Gaertn. [Nelumbonaceae; Nelumbinis semen]}, Bohe {*Mentha canadensis* L. [Lamiaceae; Menthae haplocalycis herba]}, and Chuncai {*Brasenia schreberi* J.F.Gmel. [Cabombaceae] (Wang et al., 2017)} into Chizhi for soup was used to alleviate irritation, raving, and dizziness in Xiao-Ke patients. The ratio of the original materials has been described in *Sheng Ji Zong Lu*, most of these Chinese Yao-Shan are made into congee, soup or juice. However, when using theses Yao-Shan for Xiao-Ke, their dosage should be strictly controlled, for example, Fengmi in Ou Mi Jiang Fang, which has high levels of fructose and glucose, may cause hyperglycemia.

*Yin Shan Zheng Yao*, written by Hu Sihui in the *Chinese Yuan Dynasty*, described that Luobo Zhou, Yeji Geng (Yeji refers to pheasant), Boge Geng (Boge refers to white pigeon), Liyu Tang {Liyu refers to *Cyprinus carpio* L. [Cyprinidae]}, Xiaomai Zhou, Damai, Qing-Liang-Mi, Qing-Xiao-Dou, Hui-Hui-Dou {which might refer to the seeds of *Cicer arietinum* L. [Fabaceae], or *Pisum sativum* L. [Fabaceae]}, bamboo shoot, watermelon, Lutou, the meat of yellow hen, beef, and the cheese from milk of cow, were the representative Yao-Shan of TCM used for Xiao-Ke. Most of these have been reported in the earlier Yao-Shan literature. The antioxidant-rich extracts from the seeds and sprouts of *Cicer arietinum* L. [Fabaceae] (250 mg/kg) mitigate starch-induced postprandial glycemic spikes in rats (Tiwari et al., 2013). The chickpea {*Cicer arietinum* L. [Fabaceae]} has the effect of lowering blood glucose in healthy human volunteers (Panlasigui et al., 1995). Thus, it is recommended for the list of foods for diabetics and hyperlipidemics in the Philippines (Panlasigui et al., 1995).

*Ben Cao Yue Yan* described about 30 kinds of Chinese Yao-Shan for Xiao-Ke, such as Sumi, Qing-Liang-Mi, Damai, Lüdou, Luobo, bamboo shoots, Tugua, Chixiaodou, the boiled juice (or soup) of Chinese chestnut, the meat of pheasant or yellow hen, the bones of rabbit, Tianluo, Jiaobai {the succulent stem of *Zizania latifolia* (Griseb.) Turcz. ex Stapf [Gramineae]}, Juruo {the present name is “Moyu”; it may refer to the tuber of “*Amorphophallus konjac* K.Koch [Araceae]” or “*Amorphophallus kiusianus* (Makino) Makino [Araceae]” (Jiang, 1974; Cui et al., 1989; Li and Long, 1989; Cui et al., 1994)}, Muer {the sporophore of *Auricularia auricula* (L.ex Hook.) Underw. [Auriculariaceae]}, Pin {*Marsilea quadrifolia* L. [Marsileaceae] (Wang, 1985; Yang and Huang, 2017; Liu, 2020)}, Puruo {the rhizome of *Typha domingensis* Pers. [Typhaceae]}, the root bark of Li {*Prunus salicina* Lindl. [Rosaceae]}, Fuci [also as “Wuyu” or “Biqi” (Yao et al., 1996; Xu, 2018)], beef, brain and stomach of castrated bull, the meat of white cock, the white parts of excrement from chicken, Che’ao {may refer to *Meretrix meretrix* L. [Veneridae] (Zhu, 2013)}, and Bang {may refer to “*Hyriopsis cumingii* Lea [Unionidae]” (Lai and Zhu, 1986) or “*Cristaria plicata* Leach [Unionidae]” (Shu and Cheng, 2006)}, autumn dew, the cold spring water, and the brine (highly mineralized water). Fourteen Yao-Shan types have been discussed previously, and the antidiabetic effects of Muer (polysaccharides; 100 or 200 mg/kg per day for 5 weeks by oral gavage) and Juruo (soluble glucomannan from konjac, 80 mg/kg per day for 4 weeks by oral gavage) have been confirmed in HFD plus STZ-induced T2MD animal models (Chen et al., 2019; Liu N et al., 2021; Xu et al., 2021; Liu et al., 2022). The neutral polysaccharide fractions (8 g/kg in diet each day for 5 weeks), rather than the acidic polysaccharide fractions, were responsible for the hypoglycemic effects of Muer polysaccharides in genetic T2DM mice (A^y^ mutation) (Yuan et al., 1998). Importantly, konjac glucomannan (3.6 g/day for 28 days) displays an antidiabetic effect in T2MD patients (Chen et al., 2003).

*Ben Cao Gang Mu*, written by Li Shizhen in the *Chinese Ming Dynasty*, is the great materia medica literature in China. It includes 1,892 kinds of TCM. In Volume III, “Xiao-Ke” from the section of Bai Bing Zhu Zhi, contains about 187 kinds of TCM for Xiao-Ke (Lei et al., 2019). Some of these can be directly used as the diet or Yao-Shan, and many of them have already been discussed above. In Volume IX, “Zhou” from the section of Gubu includes 62 kinds of Zhou (Sun and Chen, 2012), such as Xiaomai Zhou used for Xiao Ke; Shuyu Zhou (yam gruel) made by the wheat flour and yam {the Chinese name as Shuyu or Shanyao, *Dioscorea polystachya* Turcz. [Dioscoreaceae; Dioscoreae rhizoma]} for nourishing kidney essence, strengthening intestines and stomach. *Yin Shan Zheng Yao* and *Zun Sheng Ba Jian* (which is written by Gao Lian in the *Chinese Ming Dynasty*) also recorded a Yao-Shan with the name of “Shanyao Zhou”, which was made by yam, mutton, the polished round-grained rice, and few salt, it has the function of improving Xu-Lao-Gu-Zheng. Now, yam gruel has been acted as a representative TCM diet for treating diabetes (see detail below).

Zhang Xichun (1860–1933) had reported two yam containing Yao-Shan (Yu Ye Tang and Zi Cui Yin) for Xiao-Ke in *Yi Xue Zhong Zhong Can Xi Lu*, which is an important clinical literature in China at the beginning of the 20th century. Yu Ye Tang is consisted by Shanyao, Huangqi {*Astragalus mongholicus* Bunge [Fabaceae; Astragali radix], *Astragalus membranaceus* (Fisch.) Bunge [Fabaceae; Astragali radix]}, Zhimu, Ji-Nei-Jin {*Gallus gallus domesticus* Brisson [Phasianidae; Galli gigerii endothelium corneum]}, Gegen, Wuweizi {*Schisandra chinensis* (Turcz.) Baill. [Schisandraceae; Schisandrae chinensis fructus]}, and Tianhuafen. Zi Cui Yin is consisted by Jianqi (it may refer to Huangqi), Dihuang {*Rehmannia glutinosa* (Gaertn.) DC. [Plantaginaceae; Rehmanniae radix]}, Shanyao, Shanzhuyu {*Cornus officinalis* Siebold & Zucc. [Cornaceae]}, the pancreas of pig. Zhang was good at using yam in Yao-Shan, such as “Yi Wei Shuyu Yin (a boiled drinking made only by yam)” for “Lao-Zhai-Fa-Re”; “Shuyu Zhou (yam gruel, a gruel made only by yam)” for “yin deficiency and Lao-Re”; “Shuyu Ji-Zi-Huang Zhou (the gruel made by yam and chicken yolk)” for diarrhea; “Shuyu Fuyi Zhou {the gruel made by yam and the mature seeds of *Plantago asiatica* L. [Plantaginaceae; Plantaginis semen], *Plantago depressa* Willd. [Plantaginaceae; Plantaginis semen]}” for “yin deficiency and kidney dryness”; and “Shuyu Banxia Zhou, the gruel made by yam and Banxia {*Pinellia ternata* (Thunb.) Makino [Araceae; Pinelliae rhizoma]}” for vomiting. Many of these are still wildly used today.

Recently, yam gruel (made only by boiled the homogenate of yam into water) has been extensively investigated for its role in improving diabetes. Yam gruel (0.5 g/mL; 15 mL/kg per day for 4 or 6 weeks by oral gavage) has anti-inflammatory, hypoglycemic, and hypolipidemic effects in T2DM rat models induced by high-fat/high-sugar diet plus STZ, and improves the cognitive function of T2DM rats with focal cerebral ischemia-reperfusion (I/R) injury (Chen et al., 2017; Hong et al., 2020; Lin et al., 2020; Luo Z et al., 2021; Hong et al., 2022). Mechanistically, yam gruel inhibits excessive gluconeogenesis in the liver by reducing hepatic phosphoenolpyruvate carboxykinase (PEPCK) and glucose-6-phosphatase (G6Pase) expression (Luo Z et al., 2021) and increases AMPK expression in the pancreas and skeletal muscle (Dai et al., 2021; Lin et al., 2022). Yam gruel also elevates short-chain fatty acids (SCFAs) in stool and the abundance of SCFAs-producing bacteria in single T2DM rats or the T2DM rats with focal cerebral I/R injury (Hong et al., 2020; Lin et al., 2020). SCFAs can promote colonic glucagon-like peptide-1 (GLP-1) and peptide YY (PYY) secretion via activating free fatty acid (FFA) receptor G protein-coupled receptor 43 (GPR43) (Psichas et al., 2015). GLP-1, released from gut enteroendocrine cells, controls meal-related glycemic excursions via augmenting insulin secretion and suppressing glucagon secretion (Drucker, 2018). It also inhibits food intake and gastric emptying, actions maximizing nutrient absorption while limiting weight gain (Drucker, 2018). It is speculated that the enhanced colonic expression of GPR-43 and serum levels of GLP-1 and PYY in T2DM rats by yam gruel may be the results of elevating SCFAs levels (Lin et al., 2020).

The antidiabetic effects of yam was further confirmed in HFD plus STZ-induced diabetic mice by treating with yam aqueous extract (500 or 1000 mg/kg yam aqueous extract per day for 4 weeks by oral gavage) or its active component allantoin (20 or 50 mg/kg per day for 4 weeks by oral gavage) (Ma et al., 2020). Supplement of allantoin by intravenous injection (0.5 mg/kg, 3 times per day for 3 or 5 days) had marked plasma glucose-lowering action and increased insulin sensitivity in STZ-induced diabetic rats; these actions were blocked by specific imidazoline I-2 receptors (I-2R) antagonist, BU224. The activated I-2R enhanced the release of *β*-endorphin from the adrenal gland and then promoted AMPK phosphorylation and glucose transporter type 4 (GLUT4) expression in the muscle. All these contribute to the glucose-lowering action of allantoin in STZ-induced diabetic rats (Niu et al., 2010; Lin et al., 2012). Go et al. (2015). Hyeon-Kyu Go et al. had compared the antidiabetic activity of crude yam powder, water extract of yam, and allantoin in STZ-induced diabetic rats. They found that the water extract of yam (500 mg/kg per day for 31 days by oral gavage) exerted a stronger antidiabetic effect than allantoin. The other constituents of yam (e.g., polysaccharides, dioscorin, sapogenins, choline, l-arginine, and proteins) may be responsible for the better effect of water extract of yam on diabetic rats (Go et al., 2015). Some of these contents have beneficial effects on diabetes. The Chinese yam-derived polysaccharide has a hypoglycemic effect in HFD and high sugar diet plus STZ-induced diabetic C57BL/6 mice model (200 mg/kg acidic polysaccharide daily for 5-6 weeks by oral gavage) (Feng et al., 2022), in a high-energy diet combined with dexamethasone-induced diabetic mice model (50, 100, or 150 mg/kg per day for 35 days by oral gavage) (Li et al., 2017), in alloxan-induced diabetic rat model (fed rats with nano yam polysaccharide of 50 or 100 mg/ml per day for 12 days), and in HFD-induced hyperlipidemia rat model (fed rats with nano yam polysaccharide of 50 or 100 mg/ml per day for 30 days) (Yu et al., 2020). Interventions of yam dioscorin (80 mg/kg per day by oral gavage) for 135 days reduced weight gains and improved the impaired glucose tolerance in HFD-fed C57BL/6J mice (Wu et al., 2018).

In T2DM patients, yam gruel therapy (the concentration used is 0.5 g/mL made by 150 g yam and 300 mL water, taking yam gruel daily in the morning for 12 weeks) reduces fasting blood glucose and 2-hour postprandial blood glucose with the actions of modulating gut microflora, increasing the serum levels of superoxide dismutase (SOD) and glutathione peroxidase (GSH-Px), while decreasing blood high-sensitivity C-reactive protein (hs-CRP) and serum interleukin-6 (IL-6) levels (Pang et al., 2017a; Pang et al., 2017b; Pang et al., 2017c; He et al., 2022). Moreover, treatment with yam gruel (250 mL per day in the morning, 5 times per weeks for 10 weeks) also decreases blood glucose and improves insulin resistance in female patients with gestational diabetes mellitus, while the pregnancy outcomes have no obvious influence (Zhao et al., 2021). We expect large scale clinical trials to verify the efficacy, dosage, and key molecular mechanisms of yam gruel on diabetes.

## Yao-Shan of TCM for nonalcoholic fatty liver disease

NAFLD is a metabolic disease representing the hepatic manifestation of a systemic metabolic disorder (Tilg and Effenberger, 2020). In the ancient TCM literature, there is no clear record of “NAFLD.” It belongs to different categories such as “liver stuffiness,” “pain in the subcostal region,” and “masses”. The key pathogenesis of NAFLD is generally related to the abnormal free flow of qi by the liver, dysfunction of transportation and transformation by the spleen, and loss/deficiency of the kidney essence. All of these lead to the mutual accumulation of dampness, heat, phlegm, and blood stasis in the liver, thus forming NAFLD. The TCM methods of drying dampness and resolving phlegm, promoting blood circulation and resolving blood stasis, strengthening spleen and promoting digestion, are common strategies used to treat NAFLD.

Shanzha {*Crataegus pinnatifida* Bunge [Rosaceae; Crataegi fructus], *Crataegus pinnatifida* var. *major* N.E.Br. [Rosaceae; Crataegi fructus]} has been commonly used as a traditional medicine in Asia (such as China, Korea, and Japan), and also as an essential Chinese diet (Yao-Shan) (Hussain et al., 2021). Based on the TCM theory, Shanzha can promote digestion and strengthen the spleen, circulate qi and disperse stasis, and transform turbidity and lower lipid. Therefore, Shanzha is a well example Yao-Shan of TCM for alleviating NAFLD. Clinically, long-term high-dose consumption of boiled hawthorn juice can reduce blood lipids in patients with NAFLD (Tao, 2021). The Shanzha extracts, such as vitexin [1, 10, or 20 mg/kg per day for 8 weeks by oral gavage; daily injected 6 mg/kg for 4 weeks; 200 mg/kg Shanzha extract (its key ingredient is vitexin) per day for 8 weeks by oral gavage]; procyanidins (50, 100 or 200 mg/kg per day for 8 weeks by oral gavage); and pectin pentaoligosaccharide (150 mg/kg per day for 10 weeks by oral gavage), have the abilities to attenuate obesity-induced NAFLD in HFD-fed animals partially by increasing the expression and activities of hepatic fatty acid oxidation-related enzymes, activating AMPK and autophagy, and modulating gut microbiota (Li et al., 2013; Inamdar et al., 2019; Hussain et al., 2021; Han X et al., 2022; Jiang et al., 2022). Another component corosolic acid (10 or 20 mg/kg per day for 9 weeks by oral gavage) from Shanzha alleviated carbon tetrachloride (CCl_4_) plus HFD-induced NASH in mice by inhibiting TGF-*β*1/Smad2, NF-*κ*B, and AMPK signaling (Liu G et al., 2021). According to the TCM theory, the TCM formula can increase efficiency and reduce toxicity. Therefore, Shanzha has also been used in the TCM formula for treating NAFLD and alcoholic liver disease (ALD). One Yao-Shan (20 or 30 g/kg per day for 10 weeks by oral gavage) included Shanzha, Gegen, Goji Berry, Huangjing {*Polygonatum kingianum* Collett & Hemsl. [Asparagaceae; Polygonati rhizoma], *Polygonatum sibiricum* Redouté [Asparagaceae; Polygonati rhizoma], *Polygonatum cyrtonema* Hua [Asparagaceae; Polygonati rhizoma]} (3 : 4 : 3 : 4) can alleviate insulin resistance and hepatic steatosis in CD-1 mice fed with HFD and high fructose diet (Liu et al., 2014). Shanzha, Gegen, Huangqi, Sangzhi {the twigs of *Morus alba* L. [Moraceae]}, Zexie {*Alisma orientale* (Sam.) Juz. [Alismataceae; Alismatis rhizoma], *Alisma plantago-aquatica* L. [Alismataceae; Alismatis rhizoma]}, and Danshen {*Salvia miltiorrhiza* Bunge [Lamiaceae; Salviae miltiorrhizae radix et rhizoma]} (2 : 1 : 2 : 2 : 2 : 1) compose another Chinese Yao-Shan, which (0.222, 0.667 or 2.000 g/kg per day for 4 weeks by oral gavage) protects against ALD in rats (Kwon et al., 2005). NAFLD and other metabolic diseases are always related to unhealthy dietary habits, in turn, Yao-Shan of TCM provides a unique and alternative way to solve metabolic problems from the perspective of dietotherapy.

## Yao-Shan of TCM for other metabolic disorders

In addition to diabetes and NAFLD, the Chinese have a long history of using Yao-Shan of TCM to treat other metabolic disorders, such as CCVDs, sexual dysfunction, and gout. Here, we show examples of these. *Ling Shu* ***·*** *Wu Wei* reported that “a patient with heart disease should eat Mai, mutton, apricot, and Xiebai, but avoid the salty food.” *Huang Di Shi Jin* noted that “a mustang penis is sour, salty, warm and non-toxic, it treats male penile atrophy, oligospermia.” *Shi Liao Ben Cao* showed that “making the egg white of sparrow eggs, Tianxiong {the root tuber of *Aconitum carmichaelii* Debeaux [Ranunculaceae]; powder}, and Tusizi {*Cuscuta australis* R.Br. [Convolvulaceae; Cuscutae semen], *Cuscuta chinensis* Lam. [Convolvulaceae; Cuscutae semen]; powder} as pills, take 5 pills with wine on an empty stomach, these can treat male sexual impotence, female leucorrhea, defecation adverse.” The Chinese had accumulated a lot of Yao-Shan of TCM for sexual health in ancient times. However, how they worked remains a mystery. Yao-Shan of TCM has also emerged as essential means for hyperuricemia and gout, e.g., the juice from fruit of Mei {*Prunus mume* (Siebold) Siebold & Zucc. [Rosaceae]} (500 mg/kg per day for 4 weeks by oral gavage) promotes uric acid excretion via modulating the expression of renal and intestinal urate transporters in mice with adenine-induced chronic kidney disease (Huang et al., 2022c). Based on the TCM theory, we should consider TCM diets such as nourishing yin and clearing heat for hyperuricemia and/or gout patients. However, if we do not consider the levels of purine in original materials of Yao-Shan, it obviously does not conform to modern medical theories and treatment principles. Therefore, the arrangement of Yao-Shan should not only consider the TCM theory, but also consider the characteristics of nutrients of these raw materials and even the whole Yao-Shan of TCM after cooking.

## Perspective

When discussing metabolic diseases, patients are always advised to control their diet. On the contrary, Yao-Shan of TCM will tell patients what they can eat (Figure 1). After being processed, many raw materials of the Chinese Yao-Shan have been used as drugs in TCM prescriptions. Here, their roles are drugs, not food. Under the guidance of TCM theory and cooking techniques, these raw materials can be made into food or drink, which have the characteristics of dual use of medicine and food. Here, their roles are not only effective to prevent and treat human diseases, however, most importantly, most of them are also delicious and palatable dishes. Therefore, they have always been favored by people, especially those with metabolic disorders.

The Chinese Yao-Shan (dietotherapy), an old discipline created by the Chinese, is an important part of TCM. It is indisputable that the Yao-Shan of TCM has a huge impact on human mental and physical health, not limited to CMDs. In the United States and elsewhere, governments, non-profit organizations, and companies have also pledged huge funds to investigate Food As Medicine (2023). However, there are fundamental differences in the logical construction between Yao-Shan of TCM and Western dietetics: the former is based on the idea of yin and yang and nature–human interaction and has a holistic view, while the latter regards the life characteristics of the human body as the process of chemical reaction, and the problems can be corrected and changed by chemical methods. Hence, prescribing a Chinese Yao-Shan is more complicated than prescribing a pill. The development of Yao-Shan of TCM should be guided by both the TCM theory and the cooking theory. If it is separated from the guidance of these theories, this ancient discipline will lose its source of vitality. Moreover, our lifestyles (concerning, e.g., the gut and vagina microbiome) are much different from those of ancient times. Therefore, the effects and mechanisms of this classic dietotherapy of TCM should be further confirmed in clinic and animal models.
