# Supplementary material for: Yao-Shan of traditional Chinese medicine: an old story for metabolic health
Source: Front Pharmacol. 2023 Aug 16;14:1194026. doi: 10.3389/fphar.2023.1194026 (PMC10468577; doi:10.3389/fphar.2023.1194026)
Supplement: Supplementary file 1 [file DataSheet1.docx]

**Supplementary Materials**

Supplementary materials here can help the interested readers further consult original TCM literature and understand some TCM terms. The details are as follow:

Yao-Shan of TCM (中医药膳)

Chinese Yao-Shan (中国药膳/中医药膳)

*Hou Han Shu · Lie Nv Zhuan*-74 (后汉书·列女传, 第七十四)

Ma-Wang-Dui Han Tombs (马王堆汉墓)

*Yang Sheng Fang* (养生方)

*Za Liao Fang* (杂疗方)

*Tai Chan Shu* (胎产书)

*Wu Shi Er Bing Fang* (五十二病方)

Shan Bu (膳部)

Jing Shan Si (精膳司)

Xiao-Ke (消渴)

*Qi-Bing-Lun* Section 47 (奇病论第四十七)

*Huang Di Nei Jing* ***·*** *Su Wen* (黄帝内经·素问)

invigorating qi (supplement qi, 益气)

nourishing yin (养阴)

clearing heat (清热)

promoting fluid production (generating body fluids, 生津)

*Huang Di Shi Jin* (黄帝食禁)

Yu-Shan-Zha, 鱼䱇鲊, a kind of salted fish

*Yang Sheng Yao Ji* (养生要集)

Zhang Zhan (张湛)

Yu-Zha, 鱼鲊, a kind of salted fish

*Sun Zhen Ren Shi Ji* (孙真人食忌)

Sun Simiao (孙思邈)

*San Yuan Can Zan Yan Shou Shu* (三元参赞延寿书)

Li Pengfei (李鹏飞)

*Shi Jin Fang* (食禁方)

*Ben Cao Yue Yan* (本草约言)

Xue Ji (薛己)

Luanbi (孪痹)

Wu Zhenglun (吴正伦)

*Yang Sheng Lei Yao* (养生类要)

Chen Qi (陈气)

*Shen Nong Ben Cao Jing* (神农本草经)

*Qi Juan Shi Jing* (七卷食经)

*Cui Yu Xi Shi Jing* (崔禹锡食经)

Cui Yuxi (崔禹锡)

Shi-Yin-Zi (石阴子)

Long-Ti-Zi (龙蹄子)

He-Bei-Zi (河贝子)

*Sun Zhen Ren Shi Ji* (孙真人食忌)

*Shi Liao Ben Cao* (食疗本草)

Meng Shen (孟诜)

*Meng Shen Shi Jing* (孟诜食经)

*Shi Yi Xin Jing* (食医心镜)

Zan Yin (昝殷)

*Bei Ji Qian Jin Yao Fang* (备急千金要方)

Sun Simiao (孙思邈)

*Zheng He Ben Cao* (政和本草)

Tang Shenwei (唐慎微)

*Yang Sheng Lei Zuan* (养生类纂)

Zhou Shouzhong (周守忠)

*Shi Jian Ben Cao* (食鉴本草)

Fei Boxiong (费伯雄)

*Wo Ming Lei Ju Chao* (倭名类聚抄)

Yuan Shun (源顺)

*Yi Xin Fang* (医心方)

Dan Bo Kang Lai (丹波康赖)

*Wei Sheng Mi Yao Chao* (卫生秘要抄)

Dan Bo Xing Zhang (丹波行长)

*Wan An Fang* (万安方)

Wei Yuan Xing Quan (梶原性全)

Qing-Liang-Mi (青粱米)

Chen-Su-Mi (陈粟米)

Qing-Xiao-Dou (青小豆)

Sha-Niu-Sui (沙牛髓)

*Tai Ping Sheng Hui Fang* (太平圣惠方)

Wang Huaiyin (王怀隐)

Yin Shui Bu Zhi Fang (饮水不止方)

Gualougen Geng Fang (栝蒌根羹方)

Gua-Lou-Fen Fang (栝蒌粉方)

Xinglao Zhou Fang (杏酪粥方)

Yang-Fei Geng Fang (羊肺羹方)

Huang-Ci-Ji Zhou Fang (黄雌鸡粥方)

Shen Xiao Zhu Tu Fang (神效煮兔方)

*Feng Qin Yang Lao Shu* (奉亲养老书)

Chen Zhi (陈直)

Niuru Fang (牛乳方)

Qing-Liang-Mi Yin Fang (青粱米饮方)

Qingdou Fang (青豆方)

Donggua Geng Fang (冬瓜羹方)

Lutou Fang (鹿头方)

Zhudu Fang (猪肚方)

Lugen Yin Fang (芦根饮方)

Hong Pian (洪楩)

*Shi Zhi Yang Lao Fang* (食治养老方).

*Sheng Ji Zong Lu* (圣济总录)

Song Huizong (宋徽宗)

Yanggu Tang Fang (羊骨汤方)

Tianluo Yin Fang (田螺饮方)

Lüdou Zhi Fang (绿豆汁方)

Hudou Zhi Fang (胡豆汁方)

Dihuanghua Zhou Fang (地黄花粥方)

Liangmi Zhou Fang (粱米粥方)

Kui Ji Zhi Fang (葵齑汁方)

Mai Dou Yin Fang (麦豆饮方)

Gefen Fan Fang (葛粉饭方)

Ou Mi Jiang Fang (藕蜜浆方)

Gujianggen Geng Fang (菇蒋根羹方)

Yan Chi Yin Fang (盐豉饮方)

irritation (烦躁)

raving (狂言)

dizziness (目眩)

*Yin Shan Zheng Yao* (饮膳正要)

Hu Sihui (忽思慧)

Luobo Zhou (萝卜粥)

Yeji Geng (野鸡羹)

Boge Geng (鹁鸽羹)

Liyu Tang (鲤鱼汤)

Xiaomai Zhou (小麦粥)

Hui-Hui-Dou

*Ben Cao Yue Yan* (本草约言)

*Ben Cao Gang Mu* (本草纲目)

Li Shizhen (李时珍)

Bai Bing Zhu Zhi (百病主治)

Zhou (粥)

Gubu (谷部)

Shuyu Zhou (薯蓣粥, yam gruel)

nourishing kidney essence, strengthening intestines and stomach (补肾精, 固肠胃)

*Zun Sheng Ba Jian* (遵生八笺)

Gao Lian (高濂)

Shanyao Zhou (山药粥)

Xu-Lao-Gu-Zheng (虚劳骨蒸)

Zhang Xichun [张锡纯, 1860-1933, ancestral home: Zhucheng (诸城), Shandong, China; born in: Yanshan (盐山), Hebei, China]

Yu Ye Tang, 玉液汤

Zi Cui Yin, 滋膵饮

*Yi Xue Zhong Zhong Can Xi Lu* (医学衷中参西录)

Yi Wei Shuyu Yin (一味薯蓣饮)

Lao-Zhai-Fa-Re (劳瘵发热)

yin deficiency and Lao-Re (阴虚劳热)

Shuyu Ji-Zi-Huang Zhou (薯蓣鸡子黄粥)

Shuyu Fuyi Zhou (薯蓣芣苢粥)

yin deficiency and kidney dryness (阴虚肾燥)

Shuyu Banxia Zhou (薯蓣半夏粥)

liver stuffiness (肝癖)

pain in the subcostal region (胁痛)

masses (积聚)

abnormal free flow of qi by the liver (肝失疏泄)

dysfunction of transportation and transformation by the spleen (脾失健运)

loss/deficiency of the kidney essence (肾精亏虚)

drying dampness and resolving phlegm (燥湿化痰)

promoting blood circulation and resolving blood stasis (活血化瘀)

strengthening spleen and promoting digestion (健脾消导)

promote digestion and strengthen the spleen (消食健脾)

circulate qi and disperse stasis (行气散淤)

transform turbidity and lower lipid (化浊降脂)

*Ling Shu* ***·*** *Wu Wei* (灵枢·五味)
